# What's in a Name? The Politics of ‘Precision Medicine’

**DOI:** 10.1080/15265161.2018.1431324

**Published:** 2018-04-05

**Authors:** Sarah Chan, Sonja Erikainen

**Affiliations:** University of Edinburgh

In the target article, Kraft and colleagues (2018) use the term “precision medicine” to denote the ongoing shift towards large-scale, population-based network or so-called systems approaches in biomedicine. Alternative language, however, such as “personalized” and “stratified” medicine, also exists to describe these approaches. Rather than simply interchangeable terms, these different labels reflect the existence of multiple ideas about what, exactly, systems approaches entail and what their aims are in relation to health care, in turn based on different aspirations regarding the future of medicine and moral and political assumptions about the role and function of health care systems. The authors’ terminological choices, both in framing their argument in relation to the “precision medicine research enterprise” and in the library metaphor through which they choose to present this research, thus mirror and implicitly build on wider discourses embedded in the U.S. context. We argue that a more critical consciousness of the political and socioeconomic context and the discourses mobilized by “precision medicine” is needed, to make explicit the ethical foundations and underlying values that these terms and practices reflect.

Systems approaches in medicine have been widely heralded as revolutionary in academic and policy discourses. While “personalized medicine” is the term most widely used to describe systems approaches and their potential (Pokorska-Bocci et al. 2014), some have argued that the term “stratified medicine” more realistically captures what systems approaches can actually deliver—that is, medicine based on group stratification rather than personalization. The language of stratification, however, carries potentially negative associations with social stratification and racial profiling (Farrow, Swinn, and Bua 2014). Based on research with stakeholders, Juengst and colleagues (2016) argued that the term has failed to gain traction in the United States in particular due to these associations, which should be contextualized in relation to American politics of race and the long history of both racial segregation built on scientific racism, and powerful antiracist political opposition in response to this. They argue that a rhetorical “rebranding” has taken place in the United States, moving from the language of personalization toward the term “precision medicine,” epitomized by the 2015 launch of the $215 million Precision Medicine Initiative (PMI) and the “All of Us” research program (White House 2015). This terminological “rebranding” both reflects and has implications for the ethical priorities and values that are driving the development of systems approaches and related research investment in the US.

“Precision medicine” avoids what the World Health Organization (WHO) calls the “possibly overambitious” promise of “personalized medicine” (WHO 2013, 180) while still enabling policymakers to apply the rhetorical force of medicine “tailored to individuals’ lifestyles, genes, environment and preferences” (White House 2015). It avoids “stratified medicine's” associations with social stratification, while reframing the group stratification and medical profiling that systems approaches enact as ethically neutral or positive “precision.” It also enables the joining of genomics (and other -omics fields) with other ethically complex and controversial research modes including biobanking and data mining of electronic health records under the mantle of “precision,” and associations of the collation of these data-intensive research modes with notions such as “precision equipment.” It even resonates with “military precision” and concepts like “precision bombing” in contemporary American culture (Juengst et al. 2016).

The choice, in the U.S. context, of “precision medicine” to describe this form of research thus reflects policymakers’ need for public approval for policy initiatives and investment in systems approaches. It exploits the appeal of medicine focused on individuals’ needs, and also builds on public discourses around patient empowerment and autonomy. In making their argument regarding the importance of trust to the “precision medicine” research enterprise, Kraft and colleagues draw implicitly on these situated discourses without acknowledging or unpacking them.

While Kraft and colleagues emphasize trustworthiness as both an intrinsic ethical and an instrumental value, strategies applied by proponents of the PMI to attract public trust complicate their arguments focused on facilitating trust in the precision medicine research enterprise. PMI proponents have mobilized citizenship rhetoric and appealed to constructs of a shared national identity in ways reflecting particular cultural values and policy agendas of the initiative. The “All of Us” program aims to correlate a wide range of data with health outcomes and make the results widely accessible, including to “citizen scientists” and “engaged participants” (National Institutes of Health [NIH] 2015, 19–20). The aim is to “empower individuals and families to invest in and manage their health” by providing them with more “precise” medical information to modify their lifestyle and take personal control over their health (White House 2015).

“Precision medicine” is thus intertwined with empowerment discourses that build on models of citizens’ active participation in research and health care, where both are framed around a social contract model of benefits and obligations. The health care benefits to citizens are framed as an exchange for contributing to research and taking responsibility over one's own health. This mirrors broader discourses around what has been called “responsibilization” in various health care contexts (Nuffield Council of Bioethics 2010), whereby rhetoric focused on citizens’ empowerment is translating into increased individual responsibility over health and, consequently, a diminished burden of care carried by state and public health authorities.

Like the language of “precision,” emphasis on individualization carries strong resonance in American culture, where a liberal individualistic ethos is embedded in the value system, but it is also combined with nationalistic discourses around “our” shared national interests and values (as Americans) in ways that appeal to a collective national identity—“All of Us,” as the core research program is labelled. “Precision medicine” in the United States is therefore neither politically nor ethically neutral, not only as a choice of language but as the practice of precision medicine itself—the entire “research enterprise.” If we are to trust in this enterprise, we need to enquire further as to the implicit values of the system in which we are being asked to trust.

Relatedly, Kraft and colleagues seem to presume that research initiatives like the PMI and associated databanks are “a public good,” as the library metaphor implies. They state that this metaphor was chosen because it seemed to invoke “minimal biases,” while the term “biobank” elicited “potentially misleading” analogies and “confusion” including associations with financial banks and gold mines. Yet the library metaphor, deliberately selected to avoid negative connotations, is still non-neutral: It has the opposite effect of invoking positive associations and thus carries its own bias.

In fact, the realities of the U.S. health care system throw into question the extent to which research programs like the PMI are, indeed, a “public good,” undermining the empirical accuracy of Kraft and colleagues’ presumption that the associations invoked by the alternative term “biobank” necessarily imply confusion or are misleading. Unlike in most high-income countries where public health care systems are built on welfare models, including the assumption that benefits from national research efforts should be equitably distributed to citizens, the U.S. health care system is disproportionately structured by a private and employment-based health insurance model. Health care is not similarly considered a basic right, nor can national research initiatives be uncritically assumed to be “a public good,” the benefits of which would be distributed to “the public” based on equitable principles of access. Rather, despite proponents’ rhetoric of shared benefit for “all of us,” participation in PMI research can more closely resemble altruism: donating data without direct insurance (literally) that the benefits will reach the participant (Sabatello and Appelbaum 2017).

By treating patients’ concerns over inequitable access as a “spillover” effect of general mistrust in the health care system or health care providers, Kraft and colleagues frame these concerns as matters of “personal and familial experience” or as relational issues, rather than systematic institutional problems extending to research endeavors. Yet because systemic inequities both characterize the U.S. health care system and extend to the distribution of benefits from programs like the PMI, this unnecessarily trivializes or characterizes as misplaced what are in fact legitimate concerns about wider societal inequalities, closing off possibilities for a more critical approach to “precision medicine.”

Despite their valuable contribution to understanding factors influencing trust in systems medicine research, Kraft and colleagues presume and at times reproduce, rather than critically engage with or even fully acknowledge, the underlying contextual and value-laden discourses and agendas that structure “precision medicine” in the United States. The multiple labels and related terminological debate reflect competing agendas relating to what systems approaches can and should enable, what the future of medicine should look like, and thus where investment should be directed. The choice of the “precision” term and related rhetoric to carry these hopes and investment reflects the broader American political and health care context. By not critically engaging with the ethically and politically loaded discourses within which the “precision medicine” terminology and related language is embedded, Kraft and colleagues, even if unwittingly, overlook (at best) and reproduce (at worst) these discourses in ways that legitimate them. As well as considering “the histories and cultural perspectives of diverse patients,” to understand what promotes trust and constitutes trustworthiness in research, we must contextualize the research itself and account for the wider socioeconomic and political histories through which these perspectives are formed.
